# A spin-liquid with pinch-line singularities on the pyrochlore lattice

**DOI:** 10.1038/ncomms11572

**Published:** 2016-05-26

**Authors:** Owen Benton, L.D.C. Jaubert, Han Yan, Nic Shannon

**Affiliations:** 1 Okinawa Institute of Science and Technology Graduate University, Onna-son, Okinawa 904-0495, Japan

## Abstract

The mathematics of gauge theories lies behind many of the most profound advances in physics in the past 200 years, from Maxwell's theory of electromagnetism to Einstein's theory of general relativity. More recently it has become clear that gauge theories also emerge in condensed matter, a prime example being the spin-ice materials which host an emergent electromagnetic gauge field. In spin-ice, the underlying gauge structure is revealed by the presence of pinch-point singularities in neutron-scattering measurements. Here we report the discovery of a spin-liquid where the low-temperature physics is naturally described by the fluctuations of a tensor field with a continuous gauge freedom. This gauge structure underpins an unusual form of spin correlations, giving rise to pinch-line singularities: line-like analogues of the pinch points observed in spin-ice. Remarkably, these features may already have been observed in the pyrochlore material Tb_2_Ti_2_O_7_.

## Abstract (web summary)

Neutron scattering measurements of spin-ice materials contain signature pinch-point singularities, demonstrating the existence of an emergent electromagnetic gauge field. Here, the authors propose a system in which correlations manifest in pinch lines, which may have already been observed experimentally.

## Graphical abstract

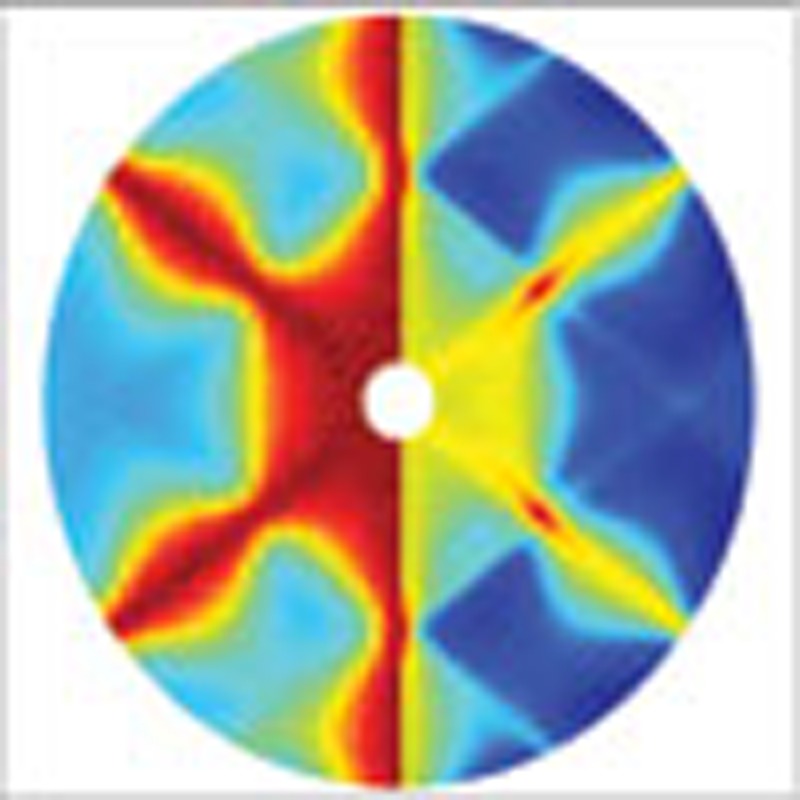

Gauge symmetries are paramount in the understanding of many of the most fundamental theories of physics. Recent decades have seen an increasing appreciation of the role of gauge theories in condensed matter physics, emerging from the long-wavelength description of the collective behaviour of electrons. Emergent gauge theories have proved particularly important in the study of spin-liquids, strongly fluctuating, disordered magnetic states, the description of which lies beyond the familiar territory of Landau theory^1^,^2^,^3^,^4^,^5^,^6^.

The use of a gauge theory to describe the fluctuations of a spin-liquid is exemplified by the case of the spin-ice materials R_2_M_2_O_7_ (R=Ho, Dy, M=Ti, Sn) (refs 7, 8). At low temperatures, the spin configurations in a spin-ice are subject to a constraint directly analogous to Gauss' law for a magnetic field and consequently may be described in terms of a gauge theory. Among the many striking consequences of this is the observation of pinch-point singularities in the magnetic neutron scattering structure factor^9^, as observed in Ho_2_Ti_2_O_7_ (ref. 10), cf. Fig. 1a. Pinch-point scattering has also been observed in the putative quantum spin-ice Tb_2_Ti_2_O_7_ (refs 11, 12, 13). However, in this case, the experimental scattering shows pronounced butterfly-like features in the non-spin-flip (NSF) channel and the scattering in the spin-flip (SF) channel shows narrow arm-like features extending along the 〈111〉 directions of reciprocal space, neither of which features are predicted for a spin-ice. This raises the question of whether other types of spin-liquid may be found amongst rare-earth pyrochlore magnets.

Here we introduce a different kind of spin-liquid on the pyrochlore lattice. This spin-liquid arises on the phase diagram of a realistic model for pyrochlore magnets. As with spin-ice, the theory of this spin-liquid contains a gauge symmetry. The nature of this theory is fundamentally different to the Maxwellian theory which describes spin-ice, but just as the emergent gauge structure of spin-ice reveals itself in pinch-point scattering, so the gauge structure of this spin-liquid has striking consequences for scattering experiments. We will show that at low temperatures, this gauge structure leads to line-like singularities along the 〈111〉 directions of reciprocal space, which we dub ‘pinch lines' since they are extended versions of the pinch-points exhibited in spin-ice. This is particularly interesting in the light of neutron scattering results on the pyrochlore magnets Tb_2_Ti_2_O_7_ and Yb_2_Ti_2_O_7_, which show strong, sharpening features along the 〈111〉 directions of reciprocal space. Indeed, our theory is able to account for several features of the diffuse scattering observed in Tb_2_Ti_2_O_7_ (refs 11, 12, 13), which are unaccounted for by a theory based on a spin-ice model.

## Results

### Spin-liquid regime in a model for pyrochlore magnets

We begin with the most general, symmetry-allowed, Hamiltonian for nearest neighbour anisotropic exchange on the pyrochlore lattice^14^,^15^,^16^:


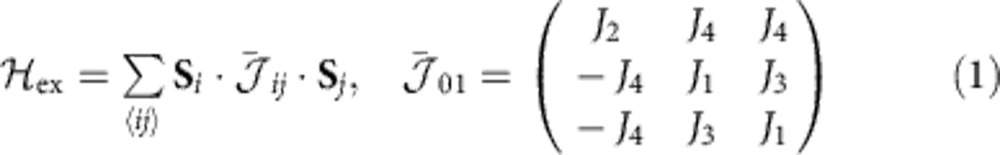


where the exchange matrix 
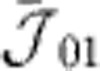
 couples nearest neighbours along the **r**_01_=(0,1,1) direction and the other exchange matrices can be generated from 
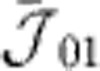
 using point group operations. As shown in refs 16, 17, it is possible to map out the entire classical ground-state phase diagram of equation (1) by an exact transcription of the Hamiltonian in terms of local fields defined on each tetrahedron^16^,^17^,^18^:


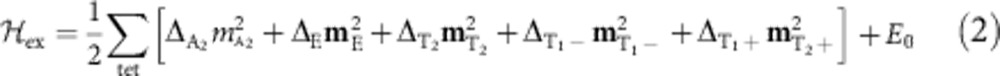


where all the coefficients Δ_*α*_≥0, *E*_0_ is the ground-state energy and the sum runs over all tetrahedra in the lattice.

The five fields 
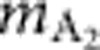
, **m**_E_, 
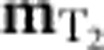
, 
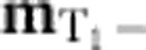
, 
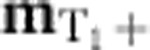
appearing in equation (2) are defined in Supplementary Table 1. They transform according to the A_2_, E, T_2_, T_1_ irreducible representations of the point group and have respective dimension 1,2,3,3 and 3. Along a line of points in parameter space the three ordered phases which respectively maximize the fields 
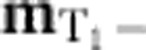
, 
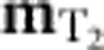
, **m**_E_ become degenerate. This line includes the point *J*_1_=*J*_2_=*J*_4_=0, *J*_3_<0 (cf. Fig. 2a). For parameter sets along this line of points we have 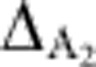
=Δ_E_=
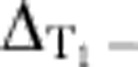
=0, 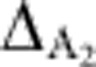, 
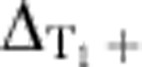
>0 and the Hamiltonian is given by


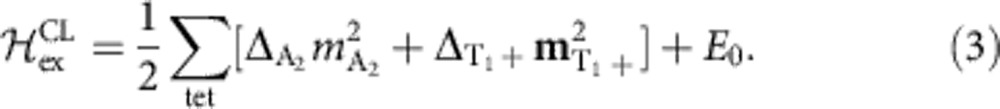


In a classical ground state of equation (3) it must be the case that


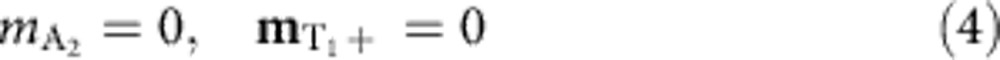


for every tetrahedron in the lattice. All of the results derived in this paper flow from the implementation of these constraints. These provide an exact description of the classical ground states along the line in parameter space where the three phases in Fig. 2a are degenerate. Observing the consequences of these constraints does not, however, require precise fine-tuning of the Hamiltonian to equation (3). These constraints will also dominate the physics at finite temperatures for any choice of parameters where 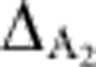, Δ_E_, 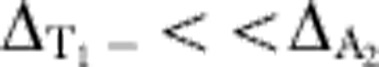, 
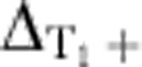
, such that energy cost of having a finite value of the fields 
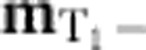
, 
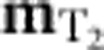
, **m**_E_ is much lower than the cost to have a finite value of 
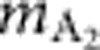
, 
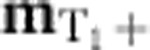
.

The constraints in equation (4) are insufficient to select an ordered ground state in themselves. In such circumstances, fluctuations may select a preferred ordered state via the order-by-disorder mechanism, but Monte Carlo simulations indicate that they fail to do so, down to temperatures 3 orders of magnitude below the scale of the bare coupling (see Fig. 2b,c). The system thus remains in a disordered but highly correlated state down to low temperature.

### Theory of the spin-liquid regime

We can understand the correlations of the spin-liquid from equation (4). The demand that the fields 
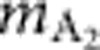
 and 
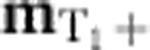
vanish everywhere leaves the fields {**m**_E_, 
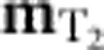
, 
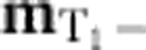
} with freedom to fluctuate in the ground state. The spatial variation of these fluctuations is constrained by the fact that neighbouring tetrahedra share a spin, therefore a fluctuation of the local fields on one tetrahedron affects the values of the local fields on the neighbouring tetrahedra. The fields **m**_E_, 
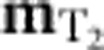
, 
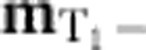
must therefore fluctuate in a correlated manner in order to avoid inducing violations of equation (4). In what follows we show how these correlated fluctuations can be understood in terms of the fluctuations of a tensor field with a continuous gauge freedom.

The constraints on the spatial variation of **m**_E_, 
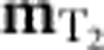
, 
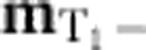
 may be obtained from the continuity of fields between *A* and *B* sublattice tetrahedra. The ground-state constraints (equation (4)) in fact imply a set of local conservation laws, on the lattice. These conservation laws in turn suggest that a coarse-graining approach can be successful in describing the fluctuations of **m**_E_, 
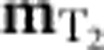
, 
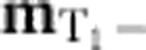
, and, unlike the global conservation laws which underpin hydrodynamic theories, these fully local conservation laws can have consequences even for short wavelength fluctuations, as we shall see. Expanding the local constraints to leading order in a gradient expansion we find


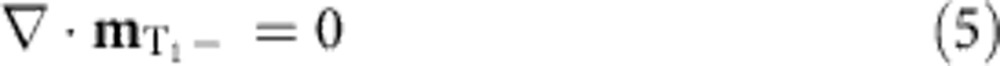



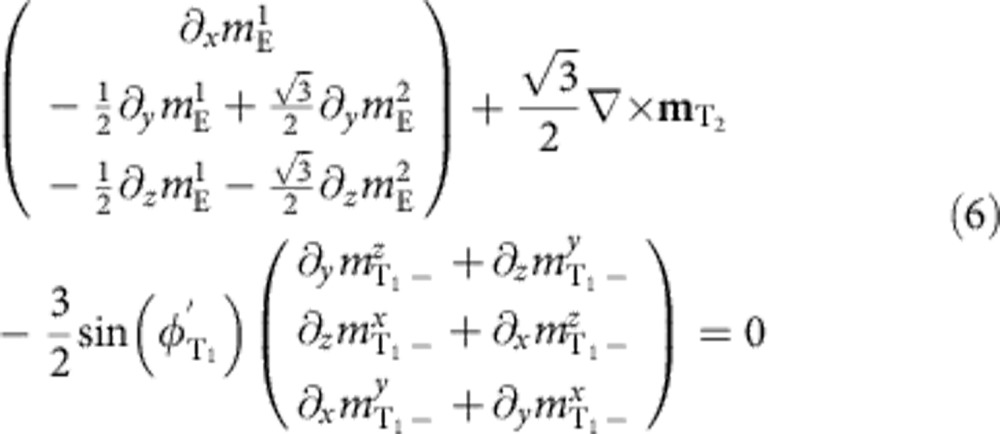


where the angle 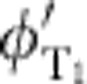 is a function of the exchange parameters, defined in the Methods section.

We wish to resolve the constraints (5) and (6) naturally using a gauge-theoretic approach. Note that since 
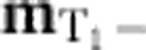
 appears in both constraints, we cannot simply introduce separate gauge fields to resolve equations (5) and (6). Instead, we incorporate the eight components of {**m**_E_, 
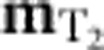
, 
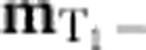
} into a traceless tensor field 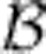:


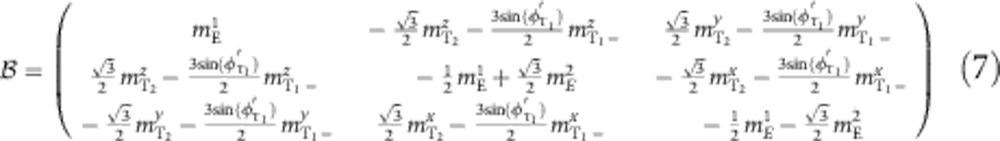


Satisfaction of equation (6), along with the condition 
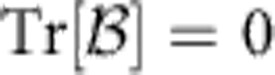
 is guaranteed by the introduction of a symmetric, tensor field 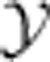 and writing


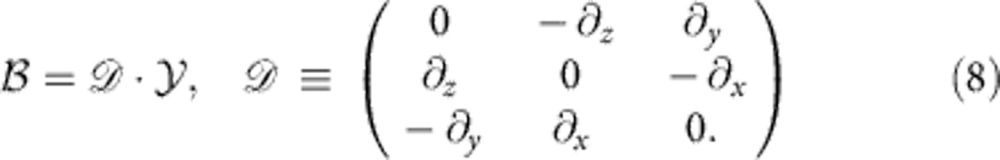


The form of the matrix gauge field 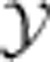 is then constrained by equation (5) which is satisfied if we take 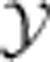 of the form


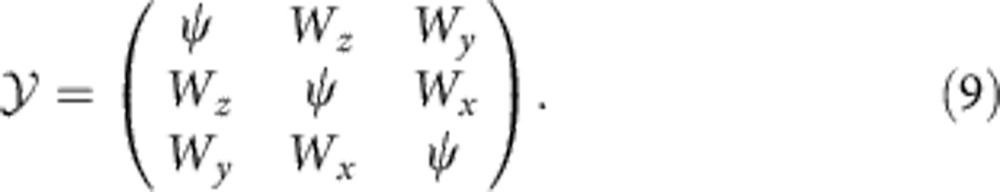


We can generate alternative forms of 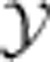 by applying Abelian gauge transformations to equation (9) of the form


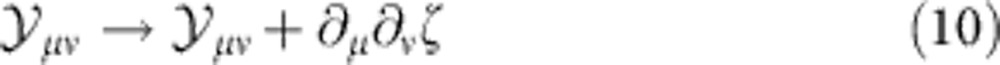


The transformations of equation (10) leave the flux matrix 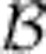, and therefore the physical spin system, unchanged. The form of 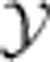 in equation (9) thus corresponds to a specific choice of gauge. The theory of the spin-liquid is therefore invariant under a group of gauge transformations 
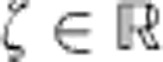
. Abelian gauge transformations of a similar form to equation (10), acting on tensor fields also appear in the linearized theory of general relativity^19^ and the theory of *S*=2 gauge fields^20^.

At low temperatures, where there are only fluctuations of the local fields **m**_E_, 
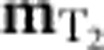
, 
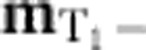
, the free energy will be controlled by the entropy of these fluctuations. Coarse-graining over some volume much larger than a unit cell but much smaller than the whole system, there will be more states available (and therefore more entropy) with small values of **m**_E_, 
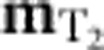
 and 
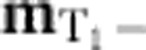
 (ref. 5). The most general symmetry-allowed Gaussian free energy describing small fluctuations of these fields, when written in terms of the tensor field 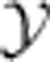, takes the form


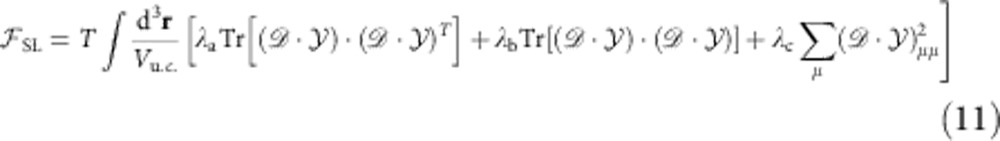


which is invariant under the gauge transformations of equation (10).

### Consequences for neutron scattering experiments

The distinctive nature of this spin-liquid, and of the theory which describes it (equation (11)), can be revealed by neutron scattering experiments. This can be seen by calculating the correlation functions of the local fields **m**_E_, 
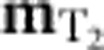
, 
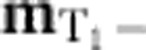
in momentum space. In addition to displaying pinch-point singularities at zone centres, these correlation functions are singular approaching any momentum **q** which is along the (*h*, *h*, *h*) directions of reciprocal space, or along any direction related by the lattice symmetry to (*h*, *h*, *h*). This contrasts with the case of the Coulombic spin-liquid which occurs in the case of spin-ice, where the correlation functions are only singular at the Brillouin zone centre. Since the fields **m**_E_, 
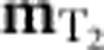
, 
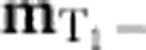
are simply linear combinations of the spins, this singular behaviour will also show up in the spin structure factor *S*(**q**), measurable in neutron scattering experiments.

In the vicinity of one of these singularities, at *T*=0, the scattering is approximated by


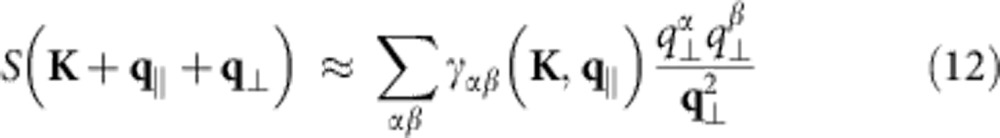


where **K** is a reciprocal lattice vector, **q**_||_ is parallel to a 〈111〉 direction and **q**_⊥_ is orthogonal to that direction. The coefficients *γ*_*αβ*_ determine the orientation of the singularity in **q**-space. Their dependence on the Brillouin zone **K** may be thought of as a form factor determining the contribution of the fluctuations of each field **m**_*λ*_ to the scattering in each Brillouin zone. The dependence on **q**_||_ is smooth and near a zone centre **K** one may write *γ*_*αβ*_(**K**, **q**_||_)≈*γ*_*αβ*_(**K**, **0**).

For 
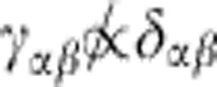
 the structure factor in the limit *q*_⊥_→0 will depend on the direction of approach and we have a singularity, along the entire 〈111〉 direction. Equation (12) has the form of a pinch-point singularity extended into a line. We therefore will refer to it as a ‘pinch-line' singularity.

These pinch lines can be observed by taking planar cuts through the scattering, which intersect these lines away from reciprocal lattice vectors (Fig. 3a). This is illustrated using a *T*=0 calculation of *S*(**q**) from the continuum theory (equation (11)) in Fig. 3b,e. For comparison, we show in Fig. 3c,f, the same quantity calculated at finite temperature within classical Monte Carlo simulation.

The simulation results show sharp features in the structure factor approaching the 〈111〉 directions, as predicted by the theory (equation (10)). There is a small broadening of these singularities, coming from the finite temperature thermal fluctuations present in the Monte Carlo simulation. These features are even more clearly visible in the correlation functions of the local fields {**m**_E_, 
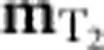
, 
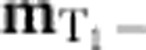
}; see Supplementary Fig. 1 and Supplementary Note 1. The presence of the pinch lines in the simulation results is a strong validation of our theory of the spin-liquid regime.

The continuum theory (equation (10)) was derived from local constraints, with associated local conservation laws, and the structure of the theory is inherited from the structure of those local constraints. This leads us to expect that the pinch-line singularities will be robust features of the spin-liquid, even at short wavelengths. We have confirmed this expectation using two independent, lattice-based calculations. Firstly, the sharpening of the scattering around the 〈111〉 directions is clearly seen in the Monte Carlo simulations in Fig. 3c,f. Secondly, we have also performed a 
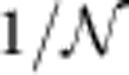
 calculation of the spin correlations along the lines of that performed for the Heisenberg model in ref. 21. This calculation also predicts pinch-line singularities along the 〈111〉 directions of reciprocal space, as shown in Fig. 3d,g. It is therefore apparent that these singularities are a robust feature of the spin-liquid, arising from the structure of its ground-state constraints, which is captured by the continuum theory derived in this work.

## Discussion

Thus far we have uncovered a spin-liquid described by a tensor field carrying a continuous gauge symmetry, arising in a particular limit of a realistic model for magnetism on the pyrochlore lattice (equation (1)). The signal feature of this spin-liquid is sharp line-like singularities along 〈111〉 directions of reciprocal space, which occur in addition to pinch-point singularities at zone centres. These pinch-line singularities are unique to the spin-liquid discussed in this paper and as such provide a very discriminating smoking-gun signature of this magnetic state. In the light of this discovery, it is interesting to consider two known pyrochlore materials, which are often discussed in the context of spin-liquid physics: Tb_2_Ti_2_O_7_ and Yb_2_Ti_2_O_7_.

Tb_2_Ti_2_O_7_ has long been a focal point for discussion of three-dimensional spin-liquid physics^22^,^23^,^24^. While equation (1) alone may not constitute a complete quantitative model for the physics of Tb_2_Ti_2_O_7_ it is interesting to compare observations on Tb_2_Ti_2_O_7_ with the phenomenology of the spin-liquid. Polarized neutron scattering experiments on Tb_2_Ti_2_O_7_ have shown evidence of singular scattering at Brilllouin zone centres, but the form of this scattering looks rather different to a typical spin-ice, especially in the non-spin-flip (NSF) channel. At the same time, the data presented in ref. 11, shows bright, narrow features extending along the 〈111〉 directions.

**Figure 1:**
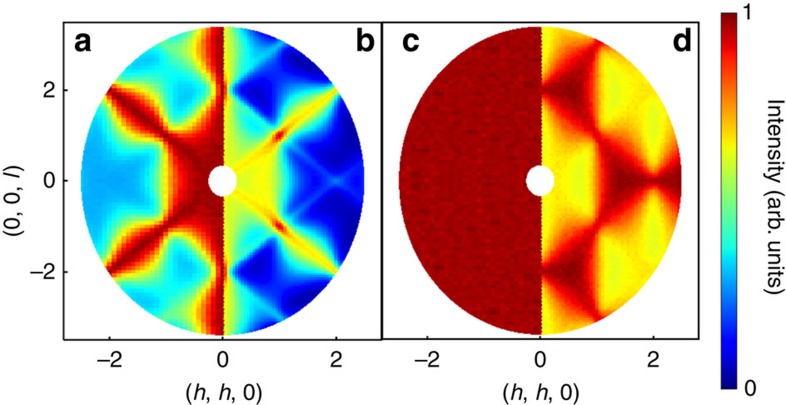
Comparison of correlations in spin-ice with those of the spin-liquid discussed in this work. Predictions for polarized neutron scattering experiments are shown in the (**a**,**b**) SF and (**c**,**d**) NSF channels, as measured by Fennell *et al*.^10^,^11^. (**a**) Prediction for spin-ice in the SF channel exhibiting pinch-point singularities. (**b**) Prediction for scattering in the SF channel in the spin-liquid discussed in this work. (**c**) Prediction for spin-ice in the NSF channel. This channel is completely featureless in a nearest neighbour model for spin-ice—as shown here—and develops smooth maxima at the zone boundaries in the presence of long range dipole interactions^10^. (**d**) Prediction for scattering in the NSF channel in the spin-liquid discussed in this work. In contrast to spin-ice, the spin-liquid discussed here exhibits singular features in both SF and NSF scattering. Results are taken from classical Monte Carlo simulation of the nearest neighbour model 
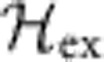
 (equation (1)), as described in the text.

As a point of comparison to these experiments, the behaviour of the structure factor *S*(**q**) in the spin-flip (SF) and non-spin-flip (NSF) channels, appropriate to a polarized neutron scattering experiment with initial polarization **n**||(1, –1, 0), is shown in Fig. 1, for the same set of exchange parameters as in Fig. 3. Narrow prominences are visible in the SF channel along the 〈111〉 directions (Fig. 1b). There are also pinch points in both channels at Brillouin zone centres. The distribution, orientation and polarization dependence of the pinch points observed in (ref. 11) is the same as that in Fig. 1b,d. In particular, we are able to reproduce the shape of the features in the NSF channel, something which cannot be done with a spin-ice-based description. The possibility that the theory described in this work could apply to Tb_2_Ti_2_O_7_ is lent weight by a recent attempt at parameterizing a pseudo-spin Hamiltonian for Tb_2+*x*_Ti_2−*x*_O_7+*y*_ (ref. 25) which places it close to the three-way phase boundary at which this spin-liquid emerges in our classical treatment.

Spin-liquid behaviour at finite temperature does not rule out the possibility of an magnetic order at lower temperature. Indeed, recent experiments have demonstrated the presence of competing ordering phenomena in Tb_2_Ti_2_O_7_, with quadrupolar^26^,^27^ and short range ordered antiferromagnetic states^12^,^28^,^29^ being observed depending on the sample stoichiometry and experimental cooling protocol. This is consistent with the nature of the spin-liquid considered in our manuscript, which sits at the confluence of many competing orders. In particular, we note that the ground-state manifold of the spin-liquid contains states consistent with the q*=(±1/2,±1/2,±1/2) order observed under field cooled conditions^12^,^28^. These states can only be connected to the other states of the spin-liquid by rotation of an 
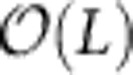
 number of spins, where *L* is the linear size of the system. This may suggest an explanation for the sensitivity to how the system is cooled—namely that field cooling may drive the system into a state from which it is hard to reach the other parts of the ground-state manifold.

**Figure 2:**
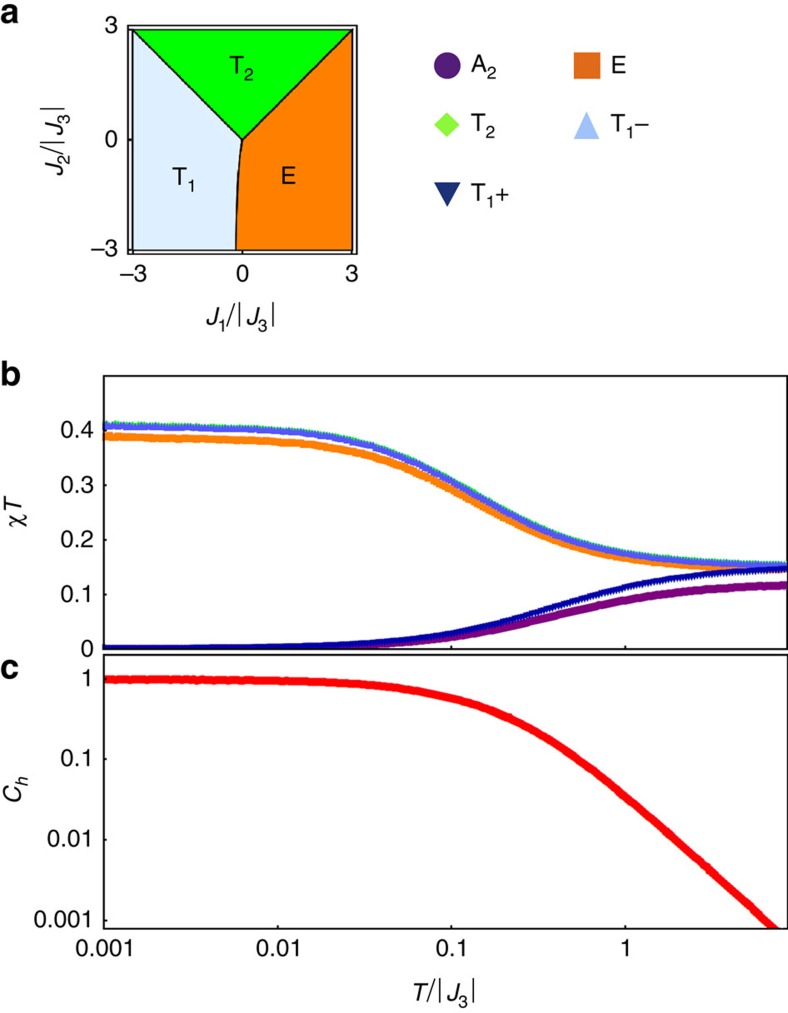
Evidence of spin-liquid behaviour from Monte Carlo simulation. (**a**) Classical ground-state phase diagram of 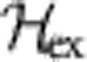 (equation (1)) for *J*_3_<0, in the plane *J*_4_=0, showing how ordered phases with symmetry T_1_, E and T_2_ meet at the point *J*_1_=*J*_2_=0 (ref. 16). (**b**) Order-parameter susceptibilities and (**c**) heat capacity calculated in classical Monte Carlo simulation of 
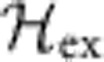
 (equation (1)), for parameters *J*_1_=*J*_2_=*J*_4_=0, *J*_3_<0. No phase transition is observed down to *T*=0.001 |*J*_3_|. Instead, the order-parameter susceptibilities of neighbouring ordered phases exhibit a Curie law crossover, characteristic of a Coulombic spin-liquid^54^. The symbols used for different symmetry channels are shown in the inset.

The combination of spin-liquid physics and prominent features in the scattering along **q**||(1, 1, 1) is also strongly reminiscent of the discussion surrounding another pyrochlore: Yb_2_Ti_2_O_7_ (refs 30, 31, 32, 33, 34). Indeed, it has recently been argued that the unusual physics of this material springs from competition between the E and T_1_ regions of the phase diagram in Fig. 2a (refs 16, 35). In this context, it is not unreasonable to imagine that the physics of the paramagnetic phase of Yb_2_Ti_2_O_7_ may be influenced by a nearby spin-liquid phase of the form described here. This provides an interesting alternative scenario to quantum spin-ice physics in that material.

**Figure 3:**
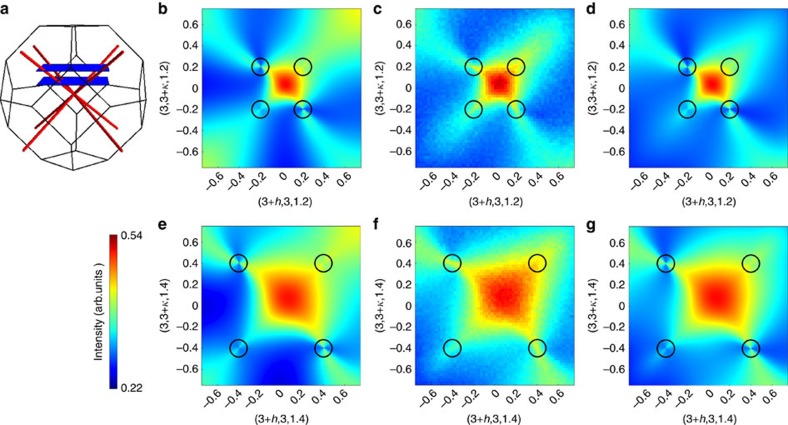
Gauge structure of the spin-liquid as revealed through pinch-line singularities. (**a**) Location of pinch-line singularities in reciprocal space. (**b**,**e**) Spin structure factor *S*(**q**) in parallel planes in reciprocal space in the Brillouin zone centred on **K**=(3,3,1), as calculated from the continuum theory (equation (11)). Singular features are visible where these planes intersect 〈111〉 directions, as indicated by the black circles in each panel. These pinch-line singularities, equation (12), are characteristic of the gauge structure of the spin-liquid. (**c**,**f**) Spin structure factor calculated in finite temperature Monte Carlo simulation, in the same regions of reciprocal space. The pinch lines appear in the simulation results as sharp features around the point where 〈111〉 directions intersect the plane. (**d**,**g**) A calculation of the structure factor made with a lattice-based 
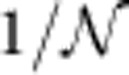
 theory, also exhibiting pinch-line singularities. The simulations were performed at a temperature *T*=0.001 K for a cluster of *N*=256,000 sites and dimensions 40*a*_0_ × 40*a*_0_ × 10*a*_0_ where *a*_0_ is the linear size of a cubic unit cell. Results were calculated for parameters *J*_1_=0.042, *J*_2_=0.122, *J*_3_=−0.118 and *J*_4_=−0.04 meV, with anisotropic *g*-tensor 
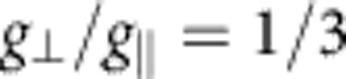
, in approximate correspondence to Tb_2_Ti_2_O_7_ (ref. 55). Since the crystal field ground state in Tb_2_Ti_2_O_7_ is a non-Kramers doublet, the finite value of *g*_⊥_ should be thought of as coming from mixing with the low-lying crystal field excitation.

One concern which arises, in any comparison with experiment, is the extent to which the validity of this theory depends on detailed, fine-tuning of parameters. At first sight, this might seem like a serious obstacle, since it is unlikely that any real material would exist exactly at the point where three different ordered phase meet. However, in practice, a moderate detuning of parameters is only likely to be important in determining the nature of the competing (classical) ground state. As long as experiments are carried out in the disordered phase, at a temperature such that violations of the constraint, equation (4), are rare, the long-wavelength physics will still be described by equation (11), and pinch lines can be observed, albeit with a finite width coming from thermal fluctuations. The robustness of pinch lines against a finite density of thermally excited violations of equation (4) is evidenced by our Monte Carlo simulations (Fig. 3c,f), which incorporate thermal excitations out of the spin-liquid ground-state manifold. Thus, at finite temperature, the signature features of the spin-liquid, including pinch lines, should remain observable for a finite region of parameter space.

Another important question is the way in which quantum fluctuations will affect the properties of the spin-liquid at low temperartures. In the one case which is fully explored, quantum spin-ice, quantum tunnelling between different spin-ice configurations stabilizes a quantum spin-liquid ground state with the same U(1) gauge structure as the parent, classical spin-liquid^36^,^37^,^38^,^39^,^40^,^41^. Meanwhile, stronger off-diagonal exchange interactions between individual spins drive the system to order at low temperatures^42^,^43^,^44^. However in both cases, classical spin-liquid behaviour is still observed over long-length scales, at finite temperature. Similarly, studies of the quantum *S*=1/2 Heisenberg model on the pyrochlore lattice find similar spin correlations^45^,^46^,^47^ to those predicted by the gauge-theoretic description of the classical problem^21^. In the present case, the nature of the ground state in the presence of quantum fluctuations is an open problem, with both ordered and quantum spin-liquid phases a realistic possibility. However, the simplest estimate of the effect of quantum fluctuations, within linear spin-wave theory, suggests that the ground state is disordered for a finite range of parameters around the classically degenerate point^16^. And, given the large entropy associated with the classical spin-liquid, it seems likely that the system will retain many of its operational features—including the extended pinch lines—at finite temperature, regardless of its quantum ground state.

The theory presented in this work provides a fundamentally different paradigm to the emergent electromagnetism known from spin-ice and possesses a gauge freedom bearing an intriguing similarity to that appearing in the linearized theory of general relativity. This leads to the possibility of a unified theory of classical spin-liquids on the pyrochlore lattice, and a classification of the above based on their associated gauge freedoms and the consequent singularities in their correlation functions. These issues will be explored further elsewhere.

The discovery of a classical spin-liquid is also a promising starting point to search for new quantum spin-liquid ground states. Previous experience suggests that most quantum spin-liquids are found in models close to a point of high classical degeneracy, and in the case of spin-ice the same gauge symmetry underpins both the classical and quantum spin-liquid states. We therefore hope that this work can open the way for even richer physics to be discovered upon the inclusion of quantum fluctuations.

In conclusion, we have demonstrated the existence of an unusual kind of classical spin-liquid phase on the pyrochlore lattice, described by the fluctuations of a tensor field with a continuous gauge freedom. The nature of this spin-liquid is revealed by pinch-line singularities in correlations which could be observed in neutron scattering experiments.

## Methods

### Decomposition of Hamiltonian in terms of local fields

It was shown in ref. 16 that the generalized model for nearest neighbour exchange on the pyrochlore lattice (equation (1)) may be exactly rewritten in terms of local fields, defined on the pyrchlore tetrahedra


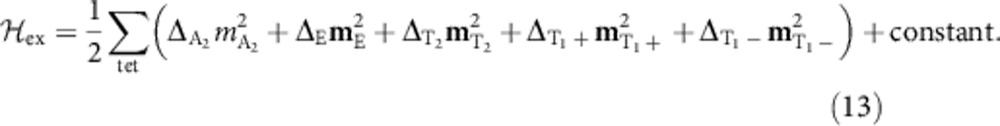


The fields are labelled by the irreducible representations of the point group according to which they transform. These fields are defined in Supplementary Table I.

The angle 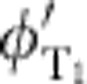 which appears in the definitions of 
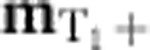
 and 
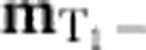
 and equation (6) is chosen such that there is no bilinear coupling between 
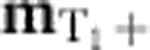
 and 
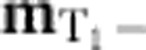
 and such that


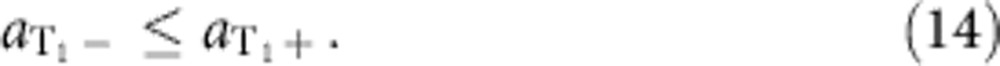


Note that this convention for the definition of the T_1_ symmetric fields is different to that chosen in ref. 16.

### Monte Carlo simulation

The classical Monte Carlo simulations used to obtain the results in Figs 1, 2, 3 are based on the Metropolis algorithm with parallel tempering^48^,^49^ and over-relaxation^50^. The spins are treated as classical vectors of fixed length |*S*_*i*_|=1/2 with local updates using the Marsaglia method^51^.

Following common practice in Monte Carlo simulations, the order-parameter susceptibilities appearing in Fig. 2 are calculated according to the following formula:


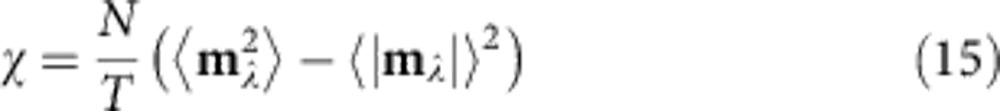


where *N* is the number of spins in the system, *T* is the temperature and *m*_*λ*_ are the local fields appearing in equation (2).

### Lattice-based calculation of the structure factor

For the purposes of comparison with the continuum theory developed in the main text, we have also performed some lattice-based calculations of the correlations in the spin-liquid regime.

These calculations follow a method which has been previously been shown successful in understanding the correlations of disordered phases of spin-ice^9^ the Heisenberg model on the pyrochlore lattice^21^,^52^, and protons in water ice^53^.

In this approach the constraints on the lengths of the spins


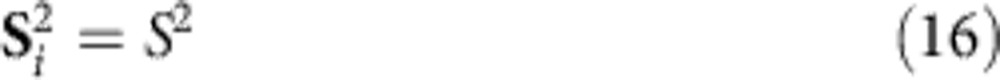


are only enforced on average


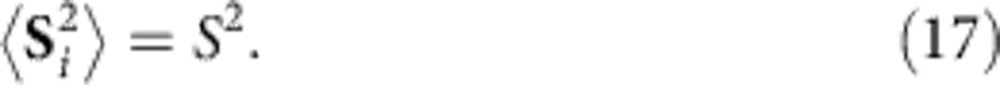


Equation (17) is enforced by means of a Lagrange multiplier *λ* added to the Hamiltonian. We write


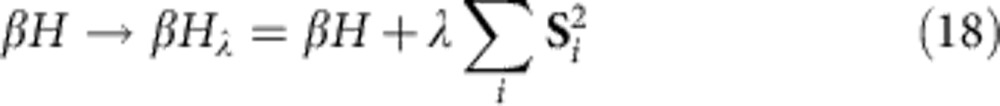


where *β* is the inverse temperature.

Using a Fourier transformation *βH*_*λ*_ may be written as


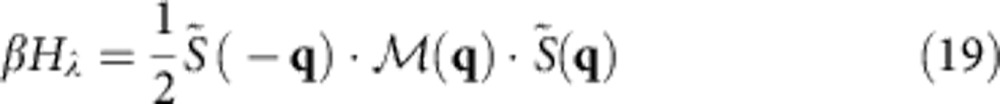


where 
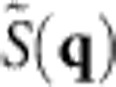
 is a 12-component vector formed from the Fourier transforms of the three spin components on each of the four sublattices.

The correlations of 
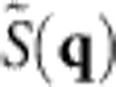
 are then


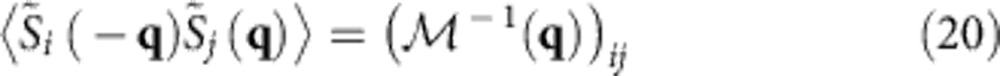


and *λ* can be chosen such that equation (17) is obeyed.

Where 
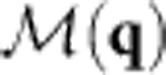
 possesses flat bands of eigenvalues at the bottom of its spectrum—as is the case in the spin-liquid regime—the limit *T*→0 of the correlation function becomes a projection matrix, projecting into the subspace described by the associated eigenvectors^53^. This projection operator can be thought of as enforcing the local ground-state constraints^9^.

It is this, zero-temperature, limit of the correlation function which is plotted in Fig. 3d,g of the main text.

The approach outlined here can be constructed as a perturbative expansion in powers of 
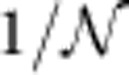
, where 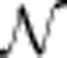 is a number of copies of the system and the spin length constraint (equation (17)) becomes


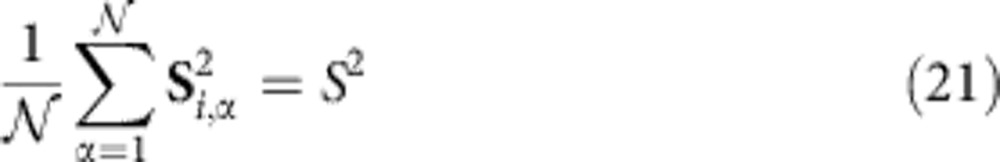


This method is described in more detail in ref. 17.

### Data availability:

This is a theoretical work. The authors declare that the data supporting the findings of this study are available within the article and its supplementary information.

## Additional information

**How to cite this article:** Benton, O. *et al*. A spin-liquid with pinch-line singularities on the pyrochlore lattice. *Nat. Commun.* 7:11572 doi: 10.1038/ncomms11572 (2016).

## Supplementary Material

Supplementary Information Supplementary Figure 1, Supplementary Table 1 and Supplementary Note 1
