# Supplementary material for: A spin-liquid with pinch-line singularities on the pyrochlore lattice
Source: Nat Commun. 2016 May 26;7:11572. doi: 10.1038/ncomms11572 (PMC4894955; doi:10.1038/ncomms11572)
Supplement: Supplementary Information — Supplementary Figure 1, Supplementary Table 1 and Supplementary Note 1 [file ncomms11572-s1.pdf]

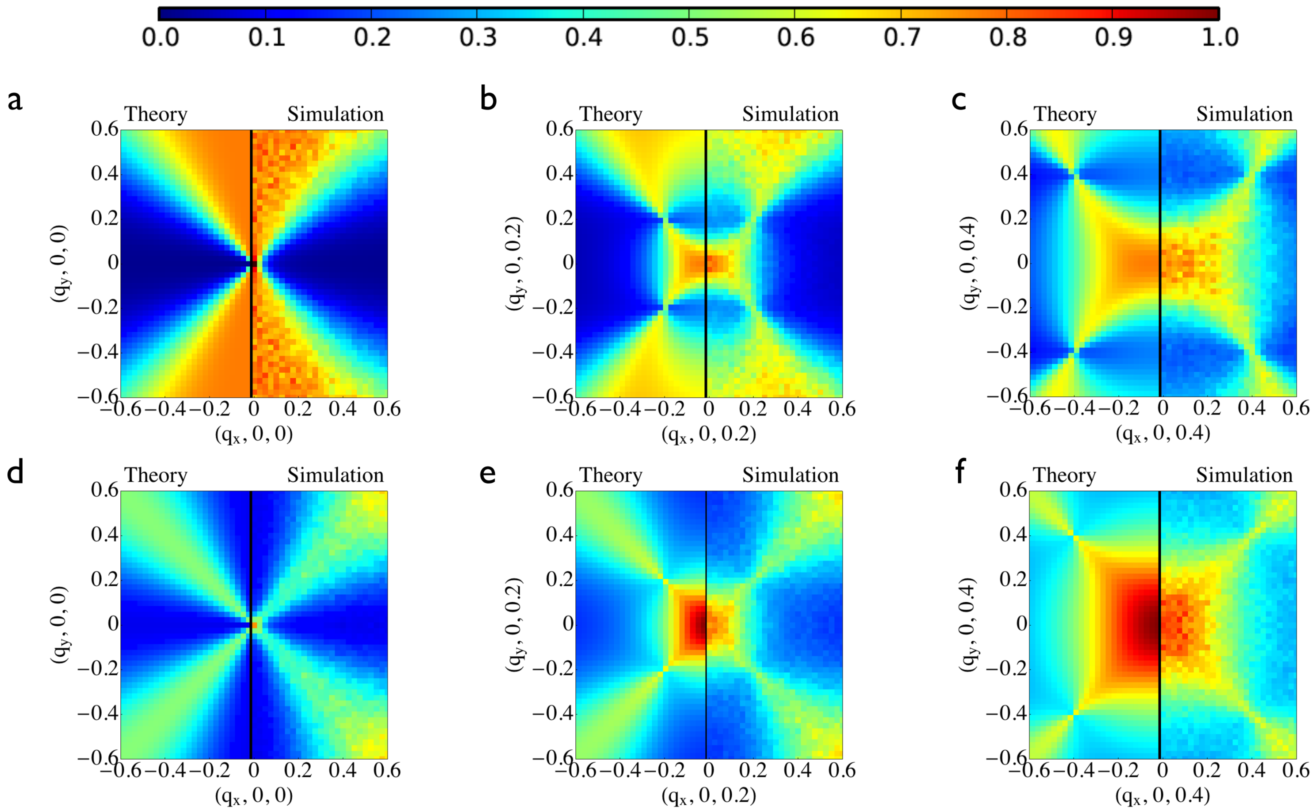

Supplementary Figure 1: Comparison of the correlation functions $\langle m^1_{\mathrm{E}}(\mathbf{q}) m^1_{\mathrm{E}}(-\mathbf{q})\rangle$, $\langle m^z_{\mathrm{T}_2}(\mathbf{q}) m^z_{\mathrm{T}_2}(-\mathbf{q})\rangle$ as calculated in the continuum field theory (left half of panels) and Monte Carlo simulation (right half of panels) of a cluster of $N = 256000$ spins at temperature $T = 0.001$ K. (a-c) Correlation function $\langle m^1_{\mathrm{E}}(-\mathbf{q}) m^1_{\mathrm{E}}(\mathbf{q})\rangle$ in parallel planes of reciprocal space $\mathbf{q} = (q_x, q_y, 0)$ (a), $\mathbf{q} = (q_x, q_y, 0.2)$ (b) and $\mathbf{q} = (q_x, q_y, 0.4)$ (c). (d-f) Correlation function $\langle m^z_{\mathrm{T}_2}(-\mathbf{q}) m^z_{\mathrm{T}_2}(\mathbf{q})\rangle$ in parallel planes of reciprocal space $\mathbf{q} = (q_x, q_y, 0)$ (d), $\mathbf{q} = (q_x, q_y, 0.2)$ (e) and $\mathbf{q} = (q_x, q_y, 0.4)$ (f). The field theory calculation is shown on a discrete grid in momentum space for the sake of comparison with the simulations which are carried out on a finite lattice with linear dimensions $L_x \times L_y \times L_z = 40 \times 40 \times 10\ a_0^3$ with periodic boundary conditions, where a_0 is the linear dimension of a cubic unit cell. The singular features found in simulation are exactly reproduced by the continuum theory [Eq. (11) of main text], with pinch-line singularities visible in both calculations where the planes of scattering cut the $\langle 111\rangle$ directions. Calculations are carried out for the set of exchange parameters J_1=0.042, J_2=0.122, J_3=−0.118, J_4=−0.04 meV, as in Fig. 3 of the manuscript.

Local field	Definition in terms of spins
m_{A_2}	$\frac{1}{\sqrt{3}}\left(S_0^x + S_0^y + S_0^z + S_1^x - S_1^y - S_1^z - S_2^x + S_2^y - S_2^z - S_3^x - S_3^y + S_3^z\right)$
$\mathbf{m}_{\mathrm{E}}$	$\begin{pmatrix} \frac{1}{\sqrt{6}}\left(-2S_0^x + S_0^y + S_0^z - 2S_1^x - S_1^y - S_1^z + 2S_2^x + S_2^y - S_2^z + 2S_3^x - S_3^y + S_3^z\right) \\ \frac{1}{\sqrt{2}}\left(-S_0^y + S_0^z + S_1^y - S_1^z - S_2^y - S_2^z + S_3^y + S_3^z\right) \end{pmatrix}$
$\mathbf{m}_{\mathrm{T}_2}$	$\begin{pmatrix} \frac{1}{\sqrt{2}}\left(-S_0^y + S_0^z + S_1^y - S_1^z + S_2^y + S_2^z - S_3^y - S_3^z\right) \\ \frac{1}{\sqrt{2}}\left(S_0^x - S_0^z - S_1^x - S_1^z - S_2^x + S_2^z + S_3^x + S_3^z\right) \\ \frac{1}{\sqrt{2}}\left(-S_0^x + S_0^y + S_1^x + S_1^y - S_2^x - S_2^y + S_3^x - S_3^y\right) \end{pmatrix}$
$\mathbf{m}_{\mathrm{T}_1\,\mathrm{ice}}$	$\begin{pmatrix} \frac{1}{\sqrt{3}}(S_0^x + S_0^y + S_0^z + S_1^x - S_1^y - S_1^z + S_2^x - S_2^y + S_2^z + S_3^x + S_3^y - S_3^z) \\ \frac{1}{\sqrt{3}}(S_0^x + S_0^y + S_0^z - S_1^x + S_1^y + S_1^z - S_2^x + S_2^y - S_2^z + S_3^x + S_3^y - S_3^z) \\ \frac{1}{\sqrt{3}}(S_0^x + S_0^y + S_0^z - S_1^x + S_1^y + S_1^z + S_2^x - S_2^y + S_2^z - S_3^x - S_3^y + S_3^z) \end{pmatrix}$
$\mathbf{m}_{\mathrm{T}_1\,\mathrm{planar}}$	$\begin{pmatrix} \frac{1}{\sqrt{6}}(-2S_0^x + S_0^y + S_0^z - 2S_1^x - S_1^y - S_1^z - 2S_2^x - S_2^y + S_2^z - 2S_3^x + S_3^y - S_3^z) \\ \frac{1}{\sqrt{6}}(S_0^x - 2S_0^y + S_0^z - S_1^x - 2S_1^y + S_1^z - S_2^x - 2S_2^y - S_2^z + S_3^x - 2S_3^y - S_3^z) \\ \frac{1}{\sqrt{6}}(S_0^x + S_0^y - 2S_0^z - S_1^x + S_1^y - 2S_1^z + S_2^x - S_2^y - 2S_2^z - S_3^x - S_3^y - 2S_3^z) \end{pmatrix}$
$\mathbf{m}_{\mathrm{T}_1 -}$	$\cos(\phi'_{\mathrm{T}_1})\mathbf{m}_{\mathrm{T}_1\,\mathrm{ice}} + \sin(\phi'_{\mathrm{T}_1})\mathbf{m}_{\mathrm{T}_1\,\mathrm{planar}}$
$\mathbf{m}_{\mathrm{T}_1,+}$	$-\sin(\phi'_{\mathrm{T}_1})\mathbf{m}_{\mathrm{T}_1\,\mathrm{ice}} + \cos(\phi'_{\mathrm{T}_1})\mathbf{m}_{\mathrm{T}_1\,\mathrm{planar}}$

Supplementary Table 1: Definition of the local fields $\mathbf{m}_\lambda$ appearing in Eq. (2) of the main text. The exchange Hamiltonian $\mathcal{H}_{\mathrm{ex}}$ [Eq. (1) of the main text] reduces to a sum of quadratic forms when written in terms of these fields. The labels $\mathrm{A}_2, \mathrm{E}, \mathrm{T}_2, \mathrm{T}_1$ refer to the irreducible representation of the point group according to which the corresponding field transforms. There are two fields which transform according to the T_1 irrep. The parameter ϕ'_{T_1} is chosen as a function of the exchange parameters to remove the symmetry-allowed coupling between these fields.

Supplementary Note 1: Correlation functions of the fields $\mathbf{m}_\lambda$

In Supplementary Figure 1 we show examples of the momentum space correlation functions of the local fields $\{\mathbf{m}_{\mathrm{E}}, \mathbf{m}_{\mathrm{T}_2}, \mathbf{m}_{\mathrm{T}_1 -}\}$, as calculated from the continuum theory developed in the manuscript (left half of panels) and from Monte Carlo simulation (right half of panels). Specifically, we show the correlation functions

$$\langle m_{\mathrm{E}}^1(\mathbf{q}) m_{\mathrm{E}}^1(-\mathbf{q})\rangle, \;\; \langle m_{\mathrm{T}_2}^{\mathrm{z}}(\mathbf{q}) m_{\mathrm{T}_2}^{\mathrm{z}}(-\mathbf{q})\rangle,$$

in three planes of reciprocal space

$$\mathbf{q} = \frac{2\pi}{a_0}(h,k,0), \;\; \mathbf{q} = \frac{2\pi}{a_0}(h,k,0.2), \;\; \mathbf{q} = \frac{2\pi}{a_0}(h,k,0.4)$$

in each case we see singular features wherever the planes cut the $\langle 111\rangle$ directions.

The Fourier transforms of the local fields are defined as

$$m_\alpha^\gamma(\mathbf{q}) = \sqrt{\frac{1}{N_{\mathrm{u.c.}}}} \sum_{\mathrm{tet}\in A} \exp[-i\mathbf{q}\cdot\mathbf{r}_{\mathrm{tet}}] m_\alpha^\gamma(\mathbf{r}_{\mathrm{tet}}) \tag{1}$$

where $N_{\mathrm{u.\,c.}}$ is the number of unit cells, the sum runs over only the 'A' sublattice of tetrahedra and $\mathbf{r}_{\mathrm{tet}}$ are the positions of the centers of those tetrahedra.

The correlation functions agree well between the continuum theory and simulation. The qualitative structure of the correlations is in agreement for all cases, although some quantitative differences are visible at shorter wavelengths. Most importantly, the Monte Carlo simulations clearly show the sharpening of the correlation functions approaching the $\langle 111\rangle$ directions, as predicted by the field theory, demonstrating that the pinch lines are a robust feature of the spin liquid regime.
